# Manipulation of JAK/STAT Signalling by High-Risk HPVs: Potential Therapeutic Targets for HPV-Associated Malignancies

**DOI:** 10.3390/v12090977

**Published:** 2020-09-03

**Authors:** Ethan L. Morgan, Andrew Macdonald

**Affiliations:** 1 Tumour Biology Section, Head and Neck Surgery Branch, National Institute on Deafness and Other Communication Disorders, NIH, Bethesda, MD 20892, USA; 2 School of Molecular and Cellular Biology, Faculty of Biological Sciences, University of Leeds, Leeds LS2 9JT, West Yorkshire, UK; 3 Astbury Centre for Structural Molecular Biology, University of Leeds, Leeds LS2 9JT, West Yorkshire, UK

**Keywords:** JAK, STAT, HPV, interferon signalling, cytokine signalling, cancer

## Abstract

Human papillomaviruses (HPVs) are small, DNA viruses that cause around 5% of all cancers in humans, including almost all cervical cancer cases and a significant proportion of anogenital and oral cancers. The HPV oncoproteins E5, E6 and E7 manipulate cellular signalling pathways to evade the immune response and promote virus persistence. The Janus Kinase/Signal Transducer and Activator of Transcription (JAK/STAT) pathway has emerged as a key mediator in a wide range of important biological signalling pathways, including cell proliferation, cell survival and the immune response. While STAT1 and STAT2 primarily drive immune signalling initiated by interferons, STAT3 and STAT5 have widely been linked to the survival and proliferative potential of a number of cancers. As such, the inhibition of STAT3 and STAT5 may offer a therapeutic benefit in HPV-associated cancers. In this review, we will discuss how HPV manipulates JAK/STAT signalling to evade the immune system and promote cell proliferation, enabling viral persistence and driving cancer development. We also discuss approaches to inhibit the JAK/STAT pathway and how these could potentially be used in the treatment of HPV-associated disease.

## 1. Introduction

Infection with high-risk human papillomaviruses (HR-HPVs) accounts for around 5% of human cancer cases worldwide, causing the majority of cervical cancers (>99%) and around 70% of oropharyngeal cancers [1]. Despite the availability of vaccines against human papillomavirus [HPV] infection, these are preventative and current treatment options are limited to platinum-based chemotherapeutics such as cisplatin, either alone or combined with radiotherapy [2]. Unfortunately, many cancers develop resistance to these drugs [3,4]; in particular, the prognosis of patients with advanced or recurrent cervical cancer is particularly poor, with a one-year survival rate of only 10–20% [5]. There are currently no specific therapeutics for HPV-associated cancers and as such, there exists a need to identify novel targets for the treatment of HPV-associated cancers.

HPVs infect the squamous and cutaneous epithelia and the expression of early viral genes create a cellular environment that promotes viral replication [6]. Additionally, HPVs have evolved efficient mechanisms to evade the immune system in order to establish persistence [7,8]. Although persistent HPV infection is a critical driver of cellular transformation, infection alone is not sufficient for malignant progression and thus other mechanisms are required [9]. HPVs manipulate a wide range of cellular processes to drive cell proliferation and viral replication, targeting the PI3K/AKT [10,11], epidermal growth factor receptor (EGFR) [12,13,14], Wnt [15] and Hippo pathways [16,17] amongst others. The viral oncoproteins E5, E6 and E7 are key in the deregulation of these pathways, promoting cell cycle progression and survival [9].

Signal Transducer and Activator of Transcription (STAT) proteins are activated by a plethora of extracellular ligands, including cytokines and growth factors, via their respective receptors [18]. STAT activation requires phosphorylation-induced homo- or heterodimerisation with other STAT proteins [19]. The tyrosine phosphorylation of STAT proteins is predominantly mediated by Janus kinases (JAKs) and Src family non-receptor tyrosine kinases, although receptor tyrosine kinases such as EGFR can also phosphorylate STATs under certain conditions [20]. STAT proteins then dimerise, translocate to the nucleus and drive gene transcription. STAT signalling regulates many diverse functions that are required for homeostasis and development in mammals [18]. Furthermore, STAT proteins have critical roles in the innate immune response (STAT1 and STAT2) and in tumour initiation and progression (STAT3 and STAT5) [20].

As a critical pathway involved in the response to pathogens, many viruses have evolved mechanisms to manipulate JAK/STAT signalling in order to evade the immune response and promote proliferation. This review will discuss how HPV modulates the JAK/STAT pathway, focusing on how this enables viral genome replication and persistence, and hence ultimately contributes towards cancer development. Additionally, we will summarise the current methods of inhibiting the JAK/STAT pathway and how these could potentially be used to treat HPV infection or HPV-associated disease.

## 2. Human Papillomaviruses

### 2.1. HPV Life Cycle

HPVs are a large family of small, double-stranded DNA viruses. At present, over 220 HPV genotypes have been identified, classified on the nucleotide sequence of the gene coding for the L1 capsid protein [21].

**Figure 1:**
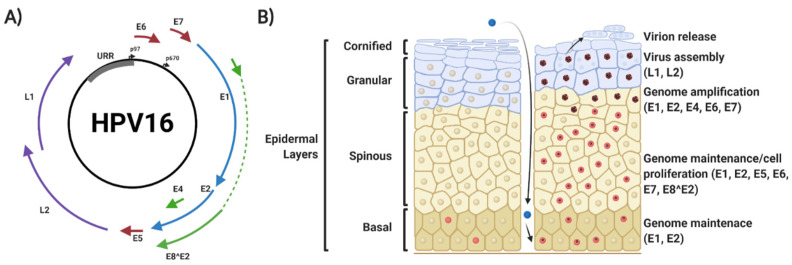
Human papillomavirus (HPV) 16 genome organisation and viral life cycle. (**A**) Organisation of the HPV16 genome, showing the relative position of the early viral genes (E1, E2, E4, E5, E6, E7 and E8^E2), the late viral genes (L1 and L2) and the upstream regulatory region (URR). The position of the early and late promoter regions is shown. (**B**) Schematic of epithelial architecture and the stages of the viral life cycle, highlighting viral genome expression profile. Details are explained in the text. Red nuclei indicate mitotically active cells. The presence of episomal HPV genomes maintains cells in a mitotically active state upon migration into the spinous layers of the epithelium, where viral amplification and late gene expression occurs. HPV virions are then released in leaky squames that are sloughed off the top layers of the epithelia. Figure created using BioRENDER.com.

The HPV genome is comprised of the early genes E1, E2, E4, E5, E6 and E7; the late genes L1 and L2; and the upstream regulatory region (URR) (Figure 1A). The URR contains the origin of replication (*ori*) site, as well as transcription factor binding sites that regulate viral transcription [22]. The viral genome also contains two promoter regions: p97 in HPV16 and 31 (p105 in HPV18) is located upstream of the E6 open reading frame (ORF) in the URR and regulates early gene transcription [23], whilst p670 in HPV16 (p811 in HPV18 and p742 in HPV31) is a differentiation-dependent promoter located within the E7 ORF and is active during productive viral replication [24].

HPVs infect the mucosal or cutaneous epithelia and viral replication is intrinsically linked to the differentiation status of the host keratinocyte [24,25,26]. Initial infection is targeted to keratinocytes in the basal epithelial layer; productive infection requires that cells remain mitotically active upon cellular differentiation for competent virus replication [27,28]. HPV gains access to target cells via microlesions generated in the skin or mucosa, and entry into the target cell requires a number of putative host receptor molecules, including heparan sulphate proteoglycans (HSPG) and α-6 integrins [29,30,31]. Binding of the major capsid protein L1 to cell receptors leads to exposure of the N-terminus of the minor capsid protein L2, allowing furin-mediated cleavage of L2 [32,33,34]. Furthermore, matrix metalloproteases (MMPs) and a disintegrin and metalloproteinase (ADAM) sheddases promote the release of HPV bound to HPSGs [35,36].

Current understanding suggests that entry may be via a macro-pinocytosis-like route that is clathrin-, caveolin-, cholesterol-, lipid raft- and dynamin-independent [37]. Viral entry also appears to be dependent on highly regulated actin dynamics and the association with CD151-containing tetraspanin-enriched microdomains [38]. Many complex alterations in the plasma membrane occur to allow viral uptake; virus induced filopodia and actin reorganization occurs via virus induced signalling pathways, such as α6β4 integrin-induced FAK and EGFR-induced PI3K/AKT/mTOR activation [35,37,39,40,41,42]. Recent data has also demonstrated an essential role for ADAM17 mediated EGFR-ERK signalling in HPV infection [43].

Disassembly of the viral capsid is initiated by acidification of the endosomal lumen, following which L1, L2 and the viral DNA traffic to the Golgi apparatus and then the endoplasmic reticulum, via an interaction between L2 and the retromer complex [44,45]. Upon delivery of the viral genome to the nucleus, it is thought to act as an autonomously-replicating episomal element and is amplified to ≈20–100 copies per cell. After initial replication, some infected daughter cells remain in the basal layer to serve as an episome reservoir (termed viral genome maintenance); here, viral gene expression is low. Other infected cells migrate into the supra-basal layer of the epidermis [6]. Here, there is a significant upregulation of viral gene expression, viral DNA replication and activation of the differentiation-dependent HPV promoter [46,47,48,49]. In uninfected epithelia, cells in the supra-basal layer exit the cell cycle to begin the terminal differentiation process; however, expression of the HPV-encoded viral oncoproteins E5, E6 and E7 prevent this cell cycle exit [14,50,51,52]. This enables amplification of the viral genome to many thousands of copies per cell [14,49,52]. As the infected cells move to the upper layers of the epithelium, they complete differentiation, enabling transit to the late stage of infection, where the late promoter is activated to drive expression of E4 and the capsid proteins L1 and L2 [53,54]. This is followed by viral capsid assembly and virion release.

### 2.2. HPV in Cancer Development

HR-HPVs have been extensively demonstrated to be oncogenic, leading to malignant transformation; however, this only occurs in a small number of cases and may take more than 20 years to develop. Studies over the past 30 years have identified that the pathogenesis of HR-HPVs is primarily driven by the activities of the virus encoded oncoproteins E5, E6 and E7. All three viral proteins have been shown to play important roles during both the viral life cycle and in tumour formation [9,55]. Much of our understanding of the functions of the oncoproteins has been derived from over-expression studies. Whilst these undoubtedly provide insight into oncogene function, it is necessary to complement them with whole virus or in vivo studies to fully appreciate their physiological relevance.

The E5 protein is a small, poorly understood viral protein, yet several studies have demonstrated that it plays a significant role during the viral life cycle. In both HPV16 and HPV31, E5 has no apparent role in viral genome maintenance or the proliferation of undifferentiated keratinocytes [56,57]. In contrast, E5 was demonstrated to play a clear role in the differentiation-dependent stages of the HPV life cycle [55]. Our data demonstrated that in HPV18, E5 is required for unscheduled DNA synthesis in supra-basal cells, but not for genome amplification or late protein expression [14], suggesting that E5 proteins from different HPV types have distinct effects during the viral life cycle. E5 has also been shown to modulate the immune response by interfering with the expression or trafficking of several critical immune receptors, including Major Histocompatibility Complex I (MHC I), MHC II and CD1d [58,59,60]. These functions contribute to the ability of HPV to avoid the innate and adaptive immune response, promoting viral persistence.

In contrast to the E6 and E7 proteins, HPV E5 proteins are weakly oncogenic and not expressed in all HPV-positive (HPV+) tumours, suggesting they may contribute to tumourigenesis in combination with E6 and E7 [61]. HPV16 E5 has been demonstrated to induce anchorage-independent cell growth and to induce mitogenic effects in several cell lines [62,63]. Furthermore, E5 was demonstrated to enhance the oncogenic abilities of HPV E7 in primary baby rat kidney cells [64]. This was validated in transgenic mouse models in which individual HPV oncoproteins, alone or in combinations, were expressed under the control of the epithelial-specific keratin-14 (K14) promoter; tumour formation was greater in mice expressing E5/E6 or E5/E7 when compared with E6 or E7 alone. Interestingly, tumour formation in mice expressing E5 alone was only observed after treatment with oestrogen, suggesting that E5 may promote, rather than initiate, tumour development [61]. This is likely to be due to several mechanisms. E5 proteins have been shown to modulate growth factor signalling; in particular, E5 promotes signalling through EGFR and this has been shown to be essential for E5-induced transformation [12,57,62,65,66]. In addition, we have demonstrated that HPV18 E5-induced EGFR signalling is essential to maintain an active cell cycle during keratinocyte differentiation, suggesting that this function of E5 may contribute to both the viral life cycle and E5-mediated tumourigenesis [14].

E6 and E7 are the primary oncogenes of HPV and have been shown to modulate a wide array of cellular proteins in order to induce cellular proliferation, avoid immune surveillance and promote cell survival [9]. Whilst most studies have looked at the effect of E6 and E7 on tumourigenesis, an essential role for both proteins in the viral life cycle has been demonstrated. Both proteins are required for stable episome maintenance, delayed keratinocyte differentiation and the hyperplasia induced upon HPV infection [67,68,69].

Both E6 and E7 play important roles in the modulation of the immune response to HPV. High risk E6 proteins, in combination with the ubiquitin ligase E6-associated protein (E6-AP), promotes the proteasomal degradation of pro-IL-1β, resulting in reduced secretion of mature IL-1β, a potent anti-viral inflammatory cytokine [70]. HPV E7 binds to Stimulator of Interferon Genes (STING), a critical DNA sensor, in an LXCXE motif dependent manner, reducing type I IFN production in response to the presence of foreign DNA [8]. E7 also binds to DNA methyltransferase 1 (DNMT1), promoting its recruitment to the promoter of chemokine (C-X-C motif) ligand 14 (CXCL14) [71]; the loss of CXCL14 expression reduces the cell surface expression of MHC I and subsequent CD8+ T cell-mediated immune responses [72].

Studies with transgenic mice show that both E6 and E7 can induce tumour formation [73,74,75,76,77,78,79,80]. Interestingly, however, E6 usually produces malignant tumours (18 out of 24), whereas E7 tends to produce benign tumours (8 out of 9) [78]. Furthermore, E6 functions in the later stages of carcinogenesis, whereas E7 is involved in tumour initiation by promoting hyperplasia. Although both proteins are sufficient to induce tumours in transgenic mice, by performing distinct functions E6 and E7 cooperate in tumour development [79].

HR-HPV E6 proteins drive many diverse processes that contribute to cancer development, often through protein-protein interactions [81]. Through binding of E6-AP, HR-HPV E6 induces the degradation of several host proteins including the tumour suppressor p53, allowing infected cells to subvert cell cycle checkpoints and avoid apoptosis. Furthermore, E6 is required for viral genome maintenance during the viral life cycle, and this is dependent on the loss of p53 expression [82]. In addition, HR-HPV E6 proteins bind and degrade a select group of PSD95/DLG/ZO-1 (PDZ) domain containing proteins via a conserved PDZ binding motif (PBM), and these functions are required for genome maintenance and the proliferation of infected keratinocytes [83]. The PBM is also required for the oncogenic properties of E6. In transgenic mice models, E6ΔPBM mice produce smaller and fewer tumours than mice expressing wild-type E6 [84]. The PBM is also required for the epithelial hyperplasia induced by HR-HPV E6 [85].

HR-HPV E7 proteins promote S phase re-entry in the differentiated strata via an ability to bind and inactivate the pocket family proteins pRb, p107 and p130 via the LXCXE motif [86]. These interactions result in release of the transcription factor E2F, causing cell cycle progression in cells that would normally be undergoing differentiation [87]. This mechanism of E7 is essential for its ability to induce epidermal hyperplasia and tumours, and may also play a role in genome maintenance [67,88]. HR-HPV E7 is also essential to induce ATM/Chk2 dependent caspase activation, which is critical for viral genome amplification upon keratinocyte differentiation [89].

## 3. Janus Kinase/Signal Transducer and Activator of Transcription (JAK/STAT) Signalling Pathways

**Figure 2:**
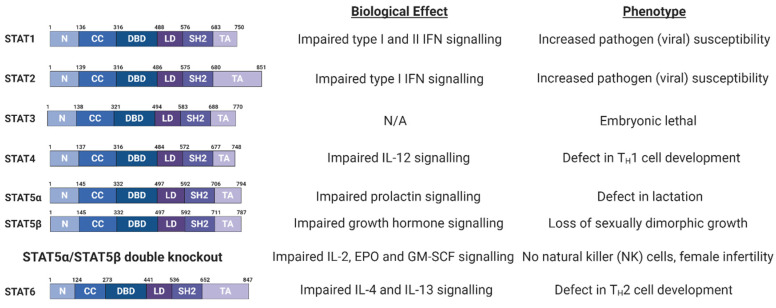
Schematic of Signal Transducer and Activator of Transcription (STAT) protein domain architecture and the biological defects and phenotypes observed in STAT family member knockout (KO) mice. IL, interleukin; IFN, interferon; EPO, erythropoietin; GM-CSF, granulocyte macrophage colony stimulating factor; N, N-terminal domain; CC, coiled coil domain; DBD, DNA-binding domain; LD, linker domain; SH2, Src Homology 2; TA, Transactivation domain [90,91,92,93,94,95,96,97]. Figure created using BioRENDER.com.

**Figure 3:**
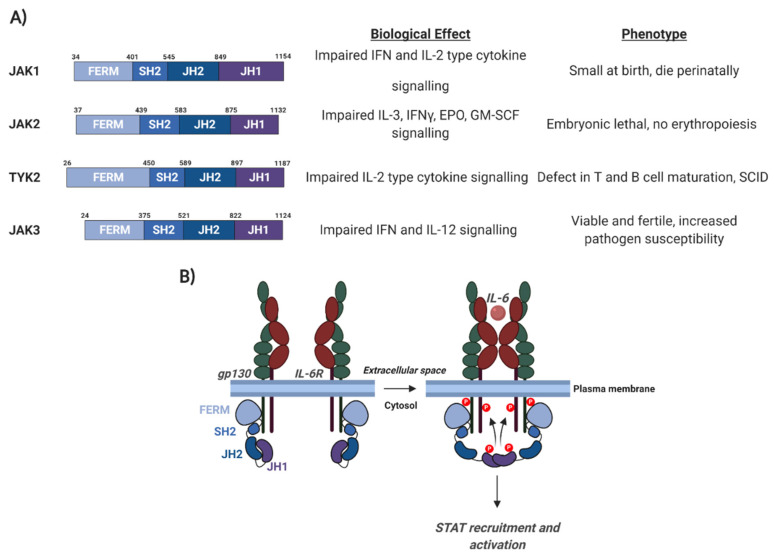
Schematic of Janus kinase (JAK) protein domain architecture and the mechanism of JAK activation. (**A**) JAK domain architecture and the biological defects and phenotypes observed in JAK family member knockout (KO) mice. gp130, glycoprotein 130; IL-6, interleukin 6; IL-6R, IL-6 receptor; FERM, 4.1 protein, Ezrin, Radixin, Moesin; SH2, Src Homology 2; JH2, JAK homology domain 2; JH1, JAK homology domain 1. (**B**) The mechanism of JAK activation. Details are discussed in the text. IL, interleukin; IFN, interferon; EPO, erythropoietin; GM-CSF, granulocyte macrophage colony stimulating factor; SCID, Severe Combined Immunodeficiency [98,99,100,101]. Figure created using BioRENDER.com.

The JAK/STAT pathway plays diverse roles that are essential for cellular homeostasis and development, relaying the signals from cytokines and growth factors to drive haematopoiesis, immune regulation, inflammation, cell proliferation and apoptosis [18]. Therefore, defects in JAK/STAT signalling can result in distinct phenotypes, as exemplified by knock out (KO) mouse studies (Figure 2 and Figure 3A).

In this review, we will discuss the roles of JAK/STAT signalling in the immune response and in cancer development and highlight how these key cellular mediators are manipulated by HPV.

### 3.1. Signal Transducer and Activator of Transcription (STAT) Family 

The STAT family of proteins were first identified as ligand-induced transcription factors in interferon (IFN) treated cells [102]. Seven STAT proteins have been identified in humans (Figure 2); STAT1 and 4 map to chromosome 2q12-33; STAT3, STAT5a and STAT5b map to chromosome 12q13-14 and STAT2 and 6 map to chromosome 17q11-22 [18].

STAT proteins range in size from 750 to 850 amino acids and all share a similar domain architecture (Figure 2; [103]). The amino-terminal domain mediates STAT protein dimerisation. Adjacent to this is a coiled coil domain, which can interact with other transcription factors and regulatory proteins. STAT proteins also contain a central DNA binding region, which recognises the consensus sequence TT(N_4-6_)AA, termed the gamma interferon activation site (GAS) [18]. In the case of type I IFN signalling, a STAT1/STAT2 heterodimer binds to the transcriptional regulator Interferon Regulatory Factor 9 (IRF9) to form the heteromeric Interferon Stimulated Gene Factor 3 (ISGF3) complex, which binds to interferon stimulated response elements (ISRE) in DNA. The Src-homology-2 (SH2) domain binds to phosphorylated tyrosine residues on other STAT proteins, mediating dimer formation [18,104]. Finally, STAT proteins contain a carboxyl-terminal transactivation domain (TAD), necessary for their transcriptional activation.

### 3.2. Janus Kinases (JAKs)

The best studied kinases necessary for STAT activation are the Janus kinases (JAKs) [105]. Four mammalian JAK proteins have been identified; JAK1, JAK2, JAK3, and tyrosine kinase 2 (TYK2), which all share considerable structural similarity (Figure 3A). JAK1, JAK2, and TYK2 are expressed in most tissue types, while expression of JAK3 is largely restricted to haemato-poietic cells [105]. The C-terminal regions of JAKs contain a JAK homology (JH) domain (JH1), in which the tyrosine kinase domain is located. The JH1 domain is preceded by a pseudo-kinase domain (JH2), which interacts with the JH1 domain to restrain kinase activity (Figure 3; [106]). In addition, JAK family members contain a four-point-one, ezrin, radixin, moesin (FERM) domain and an SH2 domain. These domains are essential for the ability of JAK proteins to bind to cell receptors, such as gp130, and recent studies have shown that the FERM domain in JAK2 can dimerise, and this is essential for JAK2 activation [107].

### 3.3. Activation of JAK/STAT Signalling

**Figure 4:**
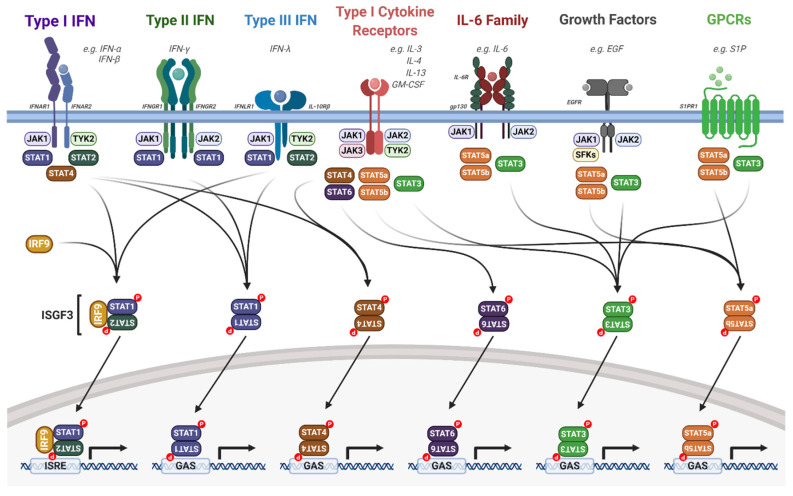
Overview of the JAK/STAT signalling pathway. Upon extracellular ligand binding to their cognate receptors, auto- and/or trans-phosphorylation of JAKs and receptor tyrosine residues occurs, acting as docking sites for STAT proteins. JAK activation leads to the phosphorylation, dimerisation and activation of STAT proteins. Dimerised STATs then translocate into the nucleus and regulate gene transcription by binding to ISRE or GAS elements. Detailed descriptions are outlined in the text. IL, interleukin; IFN, interferon; IFNAR, IFN-α receptor; IFNGR, INF-γ receptor; IFNLR, IFN-λ receptor; EGF, epidermal growth factor; EGFR, EGF receptor; GM-CSF, granulocyte macrophage colony stimulating factor; S1P, sphingosine-1-phosphate; S1PR1, sphingosine-1-phosphate receptor 1; SFKs, Src family kinases; IRF9, interferon regulatory factor 9; ISGF3, interferon stimulated gene factor 3; ISRE, interferon stimulated response element; GAS, gamma interferon activation site. Figure created using BioRENDER.com.

Although different STAT proteins have distinct biological functions, STAT activation occurs via a common mechanism. Signalling via cell surface receptors induces STAT activation via the phosphorylation of a conserved tyrosine in the carboxyl-terminus of each STAT protein [19]. In addition, STAT1, STAT3, STAT4, STAT5a and STAT5b are also phosphorylated at a conserved carboxyl-terminally located serine residue by a number of serine/threonine kinases [108]. STAT proteins are primarily activated by cytokine or growth factor signalling; however, they can also be activated by other mechanisms, such as G-protein coupled receptor (GPCR) signalling (Figure 4; [19]). Depending on the ligand and receptor, different combinations of JAKs and STATs can be activated with a high degree of specificity.

In unstimulated cells, JAK proteins are constitutively bound to cytokine receptors and in an autoinhibitory state. This is formed by the binding of the JH2 pseudo-kinase domain to the JH1 kinase domain (Figure 3B, [106]). Upon ligand binding, receptor dimerisation brings two JAKs into close proximity, allowing tyrosine phosphorylation of the cytoplasmic domains of the receptor and auto- and trans-phosphorylation of the JAKs themselves [109]. These phospho-tyrosine residues can then serve as binding sites for the SH2 domains in STAT proteins; recruitment of STATs to the receptor/JAK complex results in their phosphorylation by JAK proteins. This triggers the head-to-tail homo- or hetero-dimerisation of STATs. These dimers can then enter the nucleus to drive gene transcription by binding to specific DNA sequences (Figure 4).

As large protein complexes, STAT dimers require the nuclear pore complex (NPC) in order to efficiently shuttle into the nucleus [110]. Interestingly, the regulation of STAT nuclear transport differs between the different STAT family members. Upon tyrosine phosphorylation, a nuclear localisation sequence (NLS) in STAT1 is recognised and bound by importin-α5; importantly, the NLS can only associate with importin-α5 when STAT1 is part of an activated, phosphorylated dimer [111]. Furthermore, the affinity of STAT1 for DNA is higher than for importin-α5, allowing the release of STAT1 to bind DNA once inside the nucleus. STAT2 nuclear localisation is primarily driven by binding to IRF9; due to the constitutive NLS in IRF9, unphosphorylated STAT2-IRF9 heterodimers are shuttled to the nucleus via importin-α3, -α4 and -α7 [112,113]. However, as STAT2 contains a dominant nuclear export signal (NES), STAT2 is continuously shuttled between the cytoplasm and the nucleus. Upon phosphorylation of STAT2, it heterodimerises with phosphorylated STAT1 and is imported to the nucleus in the same manner as STAT1 [114]. Both unphosphorylated STAT3 and STAT5B, similar to STAT2, continuously shuttle between the cytoplasm and nucleus due to constitutive nuclear localisation sequences [115,116]. Furthermore, studies have demonstrated that unphosphorylated STAT3 may also influence gene expression due to its nuclear localisation [117,118].

RNA-seq studies have demonstrated that epithelial tissues widely express STAT1, STAT2, STAT3 and STAT5, as well as the kinases JAK1 and JAK2 [119]. Below, we discuss two critical aspects of JAK/STAT signalling that play important roles in HPV infection and cancer development: the regulation of antiviral immunity induced by interferons, and the induction of a hyper-proliferative environment that leads to hyperplasia.

## 4. JAK/STAT Signalling in the Immune Response

**Figure 5:**
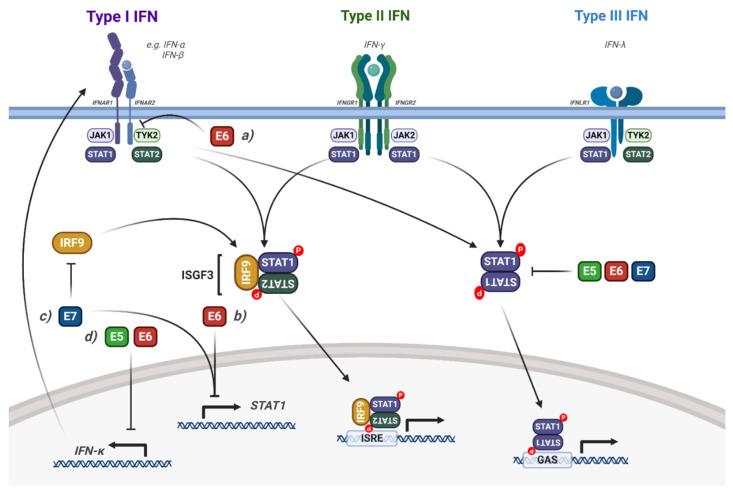
Modulation of interferon induced STAT1/2 signalling by HPV. Diagram of interferon signalling via STAT1/2; the interaction of the HPV proteins is highlighted. (**a**) HPV E6 interacts with TYK2, inhibiting IFN signalling. (**b**) HPV E6 and E7 transcriptionally repress *STAT1* expression. (**c**) HPV E7 bind to IRF9, blocking the formation of ISGF3. (**d**) HPV E5 and E6 transcriptionally repress *IFNκ* expression, inhibiting downstream STAT1 signalling. The effect of viral infection and the HPV genome is not included in the figure but is discussed in the text. IFN, interferon; IFNAR, IFN-α receptor; IFNGR, INF-γ receptor; IFNLR, IFN-λ receptor; IRF9, interferon regulatory factor 9; ISGF3, interferon stimulated gene factor 3; ISRE, interferon stimulated response element; GAS, gamma interferon activation site. Figure created using BioRENDER.com.

A key component of the immune response to viral infection is the interferon signalling pathway [120]. The JAK/STAT pathway, in particular STAT1 and STAT2, is a critical mediator of interferon signalling. Type I IFNs (such as IFNα) signal through a heterodimeric receptor complex that comprises IFNAR1 and IFNAR2; IFNγ, a type II interferon, signals through a heterodimeric transmembrane receptor that consists of the subunits IFNGR1 and IFNGR2; and the type III IFNs (such as IFNλ) signal through the receptor IFNLR1 and IL10Rβ (Figure 5; [120,121]). Interferon signalling induces an anti-viral state in the host cell that efficiently blocks viral spread and further activates a robust innate and adaptive immune response [122].

### Interaction of HPV with STAT1/2 Signalling

To establish a persistent infection, HPVs employ several mechanisms that disrupt STAT1/2 signalling and thereby inhibit the expression of interferon stimulated genes (ISGs) (Figure 5); this allows maintenance of the viral genome and continued, low-level replication in the epithelium [123]. Early microarray gene expression studies showed that overexpression of HPV16 E6, E7 or E6/E7 in immortalised keratinocytes caused a decrease in type I IFN signalling, STAT1 expression and ISG induction [124]. HR-HPV E7 can bind directly to IRF9, inhibiting the nuclear translocation of the ISGF3 complex [125]. These studies were extended by Hong et al., who showed that HPV16 and HPV31 E6 and E7 synergistically reduce STAT1 mRNA and protein expression during the virus life cycle and that this function is critical for viral genome amplification upon keratinocyte differentiation [126]. Importantly, they also showed that IFNβ treatment activated STAT1 in HPV-containing keratinocytes and reduced genome amplification, suggesting that downregulation of STAT1 is an essential, conserved function of HR-HPV E6 and E7 during the viral life cycle. Additionally, the persistence of HPV16 viral genomes also resulted in the suppression of ISGs, IFNs and STAT1 [127,128].

HPV16 can target the ISGF3 complex through additional mechanisms, most notably by downregulating the mRNA expression of *STAT2* and *IRF9* in immortalised keratinocytes (N/Tert-1 cells) [128]. This results in the decreased expression of ISGs, such as *IFIT1*, *MX1* and *OAS1*. Furthermore, the authors demonstrated that IFNβ treatment of HPV16 containing N/Tert1s restores expression of the ISGF3 complex, but not ISG expression, suggesting that HPV16 can also regulate ISG expression independently of the ISGF3 complex [128]. In this study, the viral genes E2, E6 and E7 were responsible for the downregulation of the ISGF3 complex and downstream ISG expression.

The type I IFN, IFNκ is selectively and constitutively expressed in keratinocytes and serves as a major component of the epithelial antiviral response [129]. Despite its prominence in epithelial immunity, several studies have shown that IFNκ expression is undetectable in cervical cancer cells or patient biopsies [130,131]. This loss of IFNκ expression has been linked to both the E2 and E6 proteins from HR-HPVs [132,133] and, in the case of E6, inhibition occurs in a methylation-dependent manner. Importantly, restoration of IFNκ expression increases ISG expression and generates a potent antiviral state in HPV-containing keratinocytes [134].

Recently, the HPV E5 oncoprotein has also been shown to contribute to the impairment of IFNκ signalling. Using a HPV16 mutant which results in the loss of *E5* expression, the downregulation of STAT1 and downstream ISG expression in HPV-containing keratinocytes was shown to be E5-dependent [135,136]. The authors demonstrated that this is dependent on E5-mediated methylation of the *IFNK* promoter, which is driven by activation of EGFR signalling [136]. HPV also inhibits STAT1/2 signalling via the non-receptor tyrosine kinase TYK2. Mechanistically, HPV18 E6 binds directly to TYK2 and impairs its ability to bind to IFNAR1, preventing the activation of ISGF3 and subsequent downstream ISG expression [137]. Interestingly, this function of E6 was only observed in response to IFNα, but not IFNγ [137], demonstrating a predisposition to type I IFN signalling. Finally, despite low-risk HPV11 E6 being able to bind to TYK2, it was not able to inhibit IFN signalling, suggesting that this activity may be an exclusive function of oncogenic HR-HPV E6.

## 5. STAT3 and STAT5 are Critical Drivers of HPV-Induced Malignancy

The two STAT proteins most extensively associated with cancer development are STAT3 and STAT5 [138]. Both proteins promote cancer progression by regulating the expression of cell cycle, anti-apoptotic and pro-inflammatory genes. Beyond this, STAT3 plays an important role in keratinocyte development and differentiation, suggesting it may be required during the HPV life cycle [139,140,141].

### 5.1. Interaction of HPV with STAT3 Signalling

**Figure 6:**
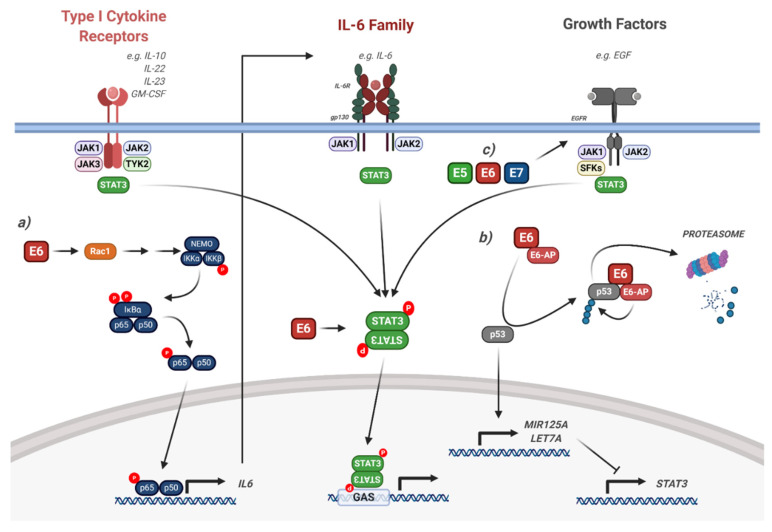
HPV modulation of the STAT3 signalling pathway. Diagram of STAT3 signalling. The interaction of the HPV proteins is highlighted. (**a**) HPV E6 induces IL-6 expression via a Rac1/NFκB signalling axis, resulting in autocrine/paracrine STAT3 signalling. (**b**) HPV E6 induces the degradation of p53, resulting in the reduction of the STAT3-targetting miRNAs miR-125a and Let-7a. (**c**) HPV E5, E6 and E7 can all induce EGFR signalling, which leads to downstream STAT3 activation. The effect of viral infection and the HPV genome is not included in the figure but is discussed in the text. IL, interleukin; EGF, epidermal growth factor; EGFR, EGF receptor; GM-CSF, granulocyte macrophage colony stimulating factor; SFKs, Src family kinases; GAS, gamma interferon activation site; E6-AP, E6-associated protein; NEMO, NFκB essential modifier; IκBα, inhibitor of NFκBα; IKK, IκB kinase. Figure created using BioRENDER.com.

STAT3 is the only family member whose genetic deletion results in embryonic lethality (Figure 2; [90]). Furthermore, STAT3 is most strongly associated with the promotion of tumour growth and immunosuppression and is a bona fide oncogene, inducing the transcription of a broad panel of genes encoding regulators of cellular proliferation (such as cyclin D1 and c-Myc), survival (such as Bcl-X_L_ and survivin) and angiogenesis (such as vascular endothelial growth factor (VEGF)) [142]. Activation of STAT3 occurs through a number of mechanisms; most commonly, this is via the IL-6 family of cytokines, which function through receptor complexes containing the gp130 co-factor (Figure 6; [109,143]). Other activators of STAT3 include type I cytokines such as IL-10 and IL-23, growth factors such as EGF and platelet-derived growth factor (PDGF) and GPCR activators such as sphingosine-1-phosphate (S1P) [144,145,146,147,148].

In keratinocytes, STAT3 has been demonstrated to have an important role in epithelial differentiation, proliferation, cell migration and survival [139,149,150,151]. We recently identified a critical role for STAT3 during the HPV18 life cycle [52]. We demonstrated that inhibition or depletion of STAT3 significantly impaired genome maintenance in undifferentiated keratinocytes in addition to differentiation-dependent viral genome amplification. We further showed that the E6 oncoprotein was required to induce both tyrosine and serine phosphorylation of STAT3, and this dual phosphorylated STAT3 was essential for productive replication [52].

Additional studies demonstrated a significant correlation between high levels of STAT3 phosphorylation and a high HPV16 viral load and integration of the viral genome, suggesting that the induction of STAT3 phosphorylation by HPV during the viral life cycle may contribute to genome integration and cancer development by an as yet unknown mechanism [152].

Several groups have reported elevated STAT3 activity in HPV-associated cancers [144,153,154,155,156,157,158,159,160,161,162,163]. In cervical cancer, STAT3 is constitutively activate and correlates with cervical disease progression [153,161,162,163]. Additionally, STAT3 phosphorylation is higher in HPV+ cervical cancers when compared with HPV-negative (HPV-) cervical cancers, suggesting that HPV actively increases STAT3 activity in these cancers [162]. Conversely, STAT3 expression and nuclear localisation is higher in HPV- head and neck squamous cell carcinoma (HNSCC), suggesting that HPV may have differential effects on STAT3 activity depending on the tissue type [164].

Our recent work, in line with other studies, clearly demonstrated a critical role for STAT3 in driving the expression of critical genes required for the proliferation and survival of HPV+ cervical cancer cells [162]. Furthermore, STAT3 has been shown to be a critical mediator of HPV E6-induced tumour formation in vivo: STAT3 knockdown by shRNA resulted in reduced tumour growth in both HPV+ cervical cancer mouse xenografts and E6-expressing HPV- cervical cancer cell xenografts [154], demonstrating that STAT3 may be an attractive therapeutic target in cervical cancer.

The mechanism of STAT3 activation induced by HPV E6 may be multi-factorial. A number of studies have shown that the microRNAs (miRNAs) miR-125a and Let-7a target STAT3 (Figure 6) [155,156,165,166]. Both miRNAs negatively correlate with STAT3 expression in cervical cancer cells. As miR-125a expression is p53-dependent, depletion of HPV E6 may induce miR-125a by limiting the proteasomal degradation of p53 [148]. In contrast, our data demonstrated that E6-induced STAT3 activation was independent of p53 degradation [52].

The EGFR is often over expressed in both HNSCC and cervical cancer and is an important driver of malignant progression [167,168]. STAT3 is a key factor downstream of EGFR signalling, and EGFR-mediated STAT3 activation is essential for skin carcinogenesis in mice [147]. However, studies showed that STAT3 activation was EGFR-independent in HNSCC cells and is instead dependent on IL-6 signalling [169]. The authors demonstrated that this is due to the fact that constitutive EGFR signalling is not commonly observed in HNSCC cell lines. Whilst investigating how HPV E6 induces STAT3 phosphorylation, we recently identified a signalling axis involving the small GTPase Rac1, the protein kinase AKT and the transcription factor Nuclear Factor kappa-light-chain-enhancer of activated B cells (NFκB) [162]. Mechanistically, Rac1, and to a lesser extent AKT, is required for the E6-mediated activation of NFκB. Furthermore, E6 induces the expression of the pro-inflammatory cytokine IL-6 in an NFκB-dependent manner and in turn, IL-6 induces the autocrine and paracrine phosphorylation of STAT3. In a subsequent study, we demonstrated that the STAT activating kinase JAK2 is essential for the activation of both STAT3 and STAT5 [163]. Other studies have demonstrated that the oncostatin M receptor (OSMR) is frequently over expressed in cervical cancer and its pro-oncogenic effects are mediated by STAT3 activation [157,159].

Although our studies and those of several other groups have demonstrated the requirement for HPV E6 in inducing STAT3 phosphorylation and activity, the other HPV oncoproteins may play minor roles. Our data demonstrated that although E6 is primarily responsible for STAT3 phosphorylation in HPV-containing keratinocytes, all three oncoproteins (E5, E6 and E7) can induce STAT3 tyrosine phosphorylation in HPV- cervical cancer cells [52]. Whilst we demonstrated that E6-mediated STAT3 phosphorylation required IL-6 autocrine/paracrine signalling, we do not know how E5 and E7 induce STAT3 phosphorylation, and whether or not this contributes to cancer development. As it has been shown that all three viral oncoproteins induce EGFR activation, it is plausible that E5 and E7 may induce STAT3 phosphorylation via the EGFR (Figure 6; [13,14,170]). Additionally, how HPV E6 activates STAT3 during an infection is not clear. Interestingly, EGFR is activated upon viral entry, suggesting that viral entry could induce EGFR-mediated STAT3 activation [42]; however, further studies are required to investigate this.

### 5.2. Interaction of HPV with STAT5 Signalling

Aberrant STAT5 activity is most commonly associated with haematological malignancies [171]. STAT5 can be activated by members of the IL-3 cytokine family, as well as other cytokines such as erythropoietin (EPO) and IL-2 family members [172]. However, in many cancers, including HNSCC, growth factors such as EGF are the primary drivers of STAT5 phosphorylation [173,174].

**Figure 7:**
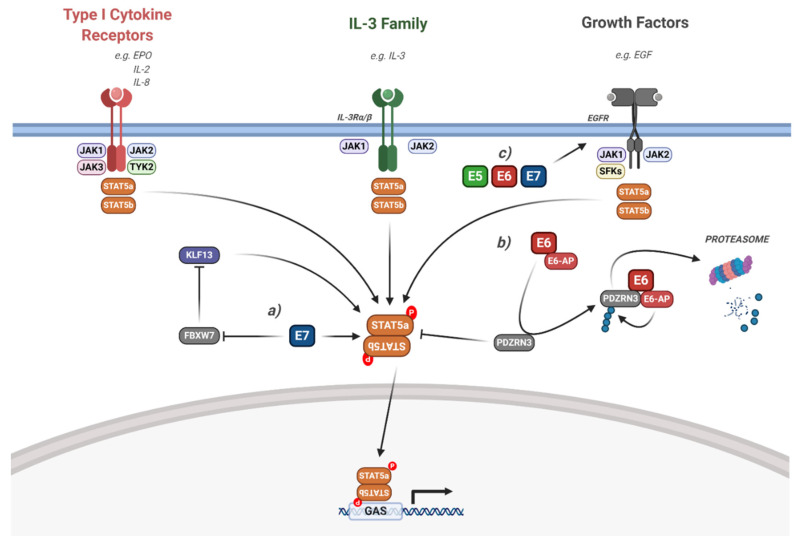
HPV modulation of the STAT5 signalling pathway. Diagram of STAT5 signalling. The interaction of the HPV proteins is highlighted. (**a**) HPV E7 activates STAT5 via the inhibition of FBWX7-induced KLF13 degradation. (**b**) HPV E6 induces STAT5 activation via the E6-AP mediated degradation of PDZRN3. (**c**) HPV E5, E6 and E7 can all induce EGFR signalling, which leads to downstream STAT5 activation. The effect of viral infection and the HPV genome is not included in the figure but is discussed in the text. IL, interleukin; EGF, epidermal growth factor; EGFR, EGF receptor; EPO, erythropoietin; SFKs, Src family kinases; KLF13, Krϋppel-like factor 13; FBXW7, F-box/WD repeat-containing protein 7; E6-AP, E6-associated protein; GAS, gamma interferon activation site. Figure created using BioRENDER.com.

The role of STAT5 in keratinocyte biology is poorly understood; however, it may play a role in epithelial differentiation [175]. In HR-HPV infected keratinocytes, E7 expression increases STAT5 tyrosine phosphorylation; active STAT5 plays a critical role in the amplification of the viral genome during the productive stage of the virus life cycle [176] (Figure 7). Interestingly, STAT5 is required for activation of the Ataxia-telangiectasia mutated (ATM) pathway in HPV-containing keratinocytes, and this is essential for viral genome amplification. Further work demonstrated that HPV-activated STAT5 could also stimulate the ataxia-telangiectasia and Rad3-related (ATR) pathway by promoting Topoisomerase IIb-binding protein 1 (TopBP1) transcription [177]. More recently, the transcription factor Krϋppel-like factor 13 (KLF13) was shown to be essential for STAT5 phosphorylation and downstream ATM activity [178]. However, how STAT5 is activated in HPV-containing keratinocytes is unclear. As for STAT3, it is plausible that EGFR activation upon viral entry may play a role in STAT5 activation during the virus lifecycle [42].

These studies demonstrate an essential role for STAT5 in driving viral replication during the HPV viral life cycle. However, whether or not it is required for the pathology of HPV-associated cancers is less clear. In head and neck cancer, STAT5b, but not STAT5a, was shown to be essential for tumour growth [173,174,179]. Importantly, this was dependent on EGFR signalling. The chemokines CCL17 and IL-2 have both been shown to induce STAT5 phosphorylation in cervical cancer cells, contributing to proliferation [180,181]. However, none of these studies demonstrated whether HPV played a role in the induction of STAT5 activity in cancer cells. Our recent study demonstrated that STAT5 phosphorylation is increased in HPV+ cervical cancer cells, indicating that STAT5 activity plays an important role in cervical cancer pathogenesis and that this is likely driven by HPV [163]. We also showed that inhibition or depletion of JAK2 decreases the phosphorylation of STAT5 in addition to STAT3, and that this inhibited the growth of HPV+ cervical cancer cells, similar to the direct inhibition of STAT5. Furthermore, we confirmed that, similar to HNSCC, this was primarily mediated by the STAT5b isoform [163]. 

Although HPV E7 was demonstrated to induce STAT5 phosphorylation in keratinocytes, both HPV E5 and E6 may also contribute. As with STAT3, activation of EGFR signalling may induce STAT5 phosphorylation in HPV-associated cancers. In line with this, we demonstrated that HR-HPV E6 induces JAK2 phosphorylation, suggesting it may also stimulate downstream STAT5 phosphorylation [52]. Furthermore, HR-HPV E6 has also be shown to be required for STAT5 phosphorylation in a PBM- and E6-AP-dependent manner by promoting the proteasomal degradation of PDZRN3 (Figure 7; [182]).

## 6. Targeting the JAK/STAT Pathway in HPV-Associated Cancers

Currently, there are no virus-specific treatments for HPV infection or HPV-associated cancers. Thus, new treatment strategies are required, and the JAK/STAT pathway offers a potential novel therapeutic avenue. As both STAT3 and STAT5 are implicated in HPV infection and HPV-associated cancers, the inhibition of these pathways may inhibit viral replication and be beneficial in the prevention or treatment of these cancers. Below, we discuss the current strategies targeting the STAT3/STAT5 signalling pathways, either directly or indirectly via inhibiting key pathways involved in their activation (Table 1).

### 6.1. Direct Targeting

#### 6.1.1. Small Molecule Inhibitors

Several STAT3 inhibitors are now entering clinical trials or pre-clinical development. Regrettably, the high toxicity of STAT3 inhibitors remains a significant challenge and thus progress has been slow [205]. Early work focused on several natural products that were identified to inhibit STAT3, such as curcumin and niclosamide [161,206]. However, these compounds have numerous off-target effects and their mechanism of action is unclear, meaning that their use in patients is unlikely. 

Building on this early work, several groups identified more specific STAT3 inhibitors by screening compound libraries, either experimentally or computationally. This led to the identification of the natural product cryptotanshinone, which selectively inhibits STAT3 by directly binding to its SH2 domain, hence preventing STAT3 dimerisation [207]. Additionally, the compound S3I-201 was also identified as a potent STAT3 inhibitor, and computational modelling suggested that this compound also directly bound to the SH2 domain to preclude dimer formation [208]. We demonstrated that both cryptotanshinone and S3I-201 potently inhibited HPV gene expression and genome amplification in keratinocytes, suggesting that abrogation of STAT3 activity can inhibit viral infection [52]. We further demonstrated that these inhibitors significantly impair the proliferation, and induce apoptosis, of HPV+ cervical cancer cells [162]. Together, these studies show that inhibition of STAT3 may be a potential therapeutic strategy in both HPV infection and HPV+ cancers.

Unfortunately, however, the relatively modest efficacy of cryptotanshinone and S3I-201 in vitro has prevented their progression into the clinic. Thus, newer STAT3 inhibitors have been developed. OPB-31121 prevents STAT3 dimerisation by binding with high affinity to the SH2 domain of STAT3 [209]. OPB-31121 has demonstrated good anti-tumour activity in leukaemia and gastric cancer cells, especially when combined with cisplatin [183,210]. However, the compound failed to produce any clinical responses in a phase I trial in advanced tumours and has been discontinued. To improve the activity of OPB-31121, OPB-111077 was developed, which is the primary metabolite of OPB-31121. This compound achieved better tissue retention and inhibited the growth of various cancers and has currently completed several phase I clinical trials in advanced cancers [184,185]. A particularly encouraging response was observed in one of the trials involving a subset of lymphomas (diffuse large B-cell lymphoma, DLBCL) and thus OPB-111077 is being assessed in further trials for clinical efficacy [184].

When compared to STAT3, much less progress has been made on the development of STAT5 inhibitors. Early studies demonstrated that the anti-psychotic drug pimozide, which is FDA approved for the treatment of several psychoses, acting by antagonising several dopamine receptors, can inhibit STAT5 activity [211]. Pimozide has shown efficacy in many types of cancer in vitro [212]; thus far, however, no clinical trials have evaluated its effect in patients. Recently, IST5-002 was identified as an inhibitor that binds to the SH2 domain of STAT5b [186]. IST5-002 inhibited STAT5 phosphorylation and reduced the growth and viability of several chronic myeloid leukaemia (CML) cell lines, including those resistant to current therapies. This compound also showed efficacy in patient-derived xenografts (PDX), demonstrating that further studies are necessary to fully appraise its potential clinical benefit [186].

An emerging class of potential cancer therapeutics are proteolysis targeting chimeras (PROTACs) that induce targeted protein degradation [213]. Recently, SD-36 was developed as a STAT3-specific PROTAC that caused STAT3 degradation by binding to the SH2 domain [187]. In this elegant study, SD-36 demonstrated excellent specificity and potency in leukaemia and lymphoma cells and induced long-lasting tumour regression in a mouse model. Furthermore, SD-36 was well tolerated in mice and showed low toxicity, suggesting that the clinical development of SD-36 and other STAT3-specific PROTACs warrants further investigation [187].

#### 6.1.2. Nucleotide Therapeutics Targeting STAT3/STAT5

Given that no small molecule inhibitors targeting STAT proteins have to date been approved for clinical use, novel methods of targeting these proteins are being investigated. Several of these methods are nucleic acid based and function by directly targeting the STAT3 mRNA, acting as a DNA-binding decoy or destabilising STAT dimers [214].

As activated STAT proteins bind to specific DNA sequences, the utilisation of so-called decoy oligonucleotides allows the ‘sponging’ of activated STAT3, inhibiting its activity [215]. To exploit this, a short, double-stranded oligonucleotide was designed based on the STAT3-binding site in the FOS gene [216]. The authors showed that this decoy potently inhibited STAT3 activity and proliferation in HNSCC cells [216]. Additional studies demonstrated the efficacy of this STAT3 decoy oligonucleotide in several cancer types, and also illustrated a benefit in overcoming EGFR inhibitor resistance [217,218]. One issue with these decoy oligonucleotides is difficulty in systemic administration; to overcome this, the authors linked the oligonucleotide strands using hexa-ethylene-glycol spacers to allow for intravenous injection. Importantly, the decoy oligonucleotide retained its potent effects on STAT3 activity and could inhibit tumour growth of HNSCC in vivo [216]. Decoy oligonucleotides have also been identified for STAT5, inhibiting the growth of CML cell lines [189]; however, in vivo studies are yet to be performed.

Another method of repressing STAT3 signalling involves the direct inhibition of STAT3 expression using antisense oligonucleotides, resulting in the degradation of STAT3 mRNA [219]. Early versions were modified with 2′-O-methyl or 2′-O-methoxyethyl moieties to enhance stability. Treatment with antisense oligonucleotides resulted in decreased STAT3-dependent gene expression is several cancer cell lines [220]; furthermore, inhibition of tumour growth was observed in mouse xenografts of prostate [221].

The second generation STAT3 antisense oligonucleotide AZD9150 has recently entered clinical trials [190]. In pre-clinical studies in lung and lymphoma cancer cells, AZD9150, which is more stable than previous iterations due to a different scaffold, decreased STAT3 expression and demonstrated significant anti-tumour activity. In a recent phase I study in DLBCL patients, AZD9150 was well tolerated and demonstrated some efficacy in a subset of heavily pre-treated patients [188]. This antisense oligonucleotide is currently being investigated in combination with checkpoint immunotherapies in DLBCL and advanced solid tumour. Recently, AZD9150, in combination with the anti-PD-1 inhibitor Durvalumab, showed promising results in a phase II trial in recurrent/metastatic (R/M) HNSCC, suggesting this may be a promising therapeutic strategy in these cancers [211].

A newer nucleotide-based therapy are the G-quartet oligodeoxynucleotides [GQ-ODNs]. GQ-ODNs are macrocycles composed of four guanosine bases that, upon hydrogen-bonding, form a poly-guanylate, tetrad-helical structure in the presence of monovalent cations such as potassium [214]. GQ-ODNs can directly destabilise STAT3 dimers by binding to the SH2 domain, inhibiting STAT3 DNA binding [191]. Xenograft studies have shown that the GQ-ODNs T40214 and T40231 significantly reduce tumour growth in prostate, breast and HNSCC models [192,222].

Together, these studies suggest that the direct targeting of STAT3 using nucleotide-based therapies can inhibit the DNA-binding ability of STAT3 and have shown promising in vivo results beyond proof-of-principle studies. However, optimisation of the potency, stability, and delivery of these nucleotide therapies is essential for enhancing their therapeutic benefits in the clinic.

### 6.2. Indirect Targeting

Due to the poor efficacy and high toxicity of STAT inhibitors in early pre-clinical trials, much research has focused on targeting upstream pathways to reduce phosphorylation and/or activation of STAT3 in tumour cells.

#### 6.2.1. Targeting IL-6 Signalling

The most prevalent mechanism of STAT3 activation in cancer is via the pro-inflammatory cytokine IL-6 [109]. Therefore, targeting of IL-6 signalling may be of therapeutic benefit in several cancers. There have been three main clinical approaches to inhibit IL-6 signalling at the ligand/receptor level: directly targeting IL-6, directly targeting the IL-6R, and targeting the IL-6/soluble IL-6R complex [109].

Several anti-IL-6 monoclonal antibodies are currently in pre-clinical development or clinical trials, the most advanced being Siltuximab, which is currently FDA approved for multicentric Castleman disease [109]. Siltuximab is currently in phase I/II trials for a number of solid tumours, including renal cell carcinoma, where it has been shown to decrease phosphorylated STAT3 levels and stabilise the disease in >50% of patients [193]. However, no clinical benefit was observed for a number of advanced tumours, including HNSCC [194]. Additional anti-IL-6 antibodies, including Olokizumab, are also in early phase clinical trials for several solid tumours, with varying efficacies [109].

Tocilizumab is an anti-IL-6R monoclonal antibody that is FDA approved for the treatment of rheumatoid arthritis (RA) [197]. Furthermore, pre-clinical studies have demonstrated efficacy in ovarian and pancreatic cancer. [198,223]. Another monoclonal antibody that targets the IL-6R, Sarilumab, is also FDA approved for RA [199].

Both of the above inhibitor classes target the classic IL-6 pathway, in which IL-6 binds to its membrane-bound receptor IL-6R. However, another form of IL-6 signalling exists, known as trans-IL-6 signalling [224]. Here, IL-6 binds to soluble IL-6R, which is produced via alternative splicing of IL6R mRNA or cleavage of membrane-bound IL-6R by disintegrin and metalloproteinase domain-containing protein 10 (ADAM10) or ADAM17. This complex can then bind to membrane-bound gp130 and activate downstream STAT3 signalling [225]. Selective inhibition of trans-signalling might be of particular value in patients whose tumours display low or no IL-6R expression; this can be achieved through the use of soluble gp130 fusion proteins. One current version, Olamkicept, is currently in phase I trials for RA and phase II trials for irritable bowel disease (IBD) [200,226].

IL-6 signalling has been demonstrated to be upregulated and associated with the activation of STAT3 in both HNSCC and cervical cancer [154,162,169,227,228]. Furthermore, we demonstrated that the HPV E6 oncoprotein upregulates IL-6 expression, resulting in the autocrine/paracrine activation of STAT3 [162]. Therefore, it is plausible that targeting the IL-6 signalling pathway may be of therapeutic benefit in HPV+ cancers.

#### 6.2.2. Targeting Janus Kinases

The key activators of STAT3 and STAT5 downstream of membrane receptors are the Janus kinases (Figure 6 and Figure 7). Many JAK inhibitors have been developed, with a heavy focus on their potential use in the treatment of chronic inflammatory and myeloproliferative disorders [229,230]. Tofacitinib primarily inhibits JAK1 and JAK3 and is an FDA-approved treatment for RA, with clinical trials ongoing for IBD [201]; ruxolitinib is selective for JAK1 and JAK2, and is approved for myelofibrosis and polycythaemia vera [202]; and parcritinib is a JAK2 inhibitor which is currently in phase II trials for myelofibrosis [204]. 

Furthermore, a number of clinically approved inhibitors have also been assessed for use in the treatment of solid tumours, although clinical data on the use of these inhibitors is currently limited. Early pre-clinical studies utilised AZD1480, a JAK1/2 inhibitor, and demonstrated STAT3 inhibition and anti-tumour activity in HPV- HNSCC PDX models [231]. More recent phase I studies have shown that ruxolitinib is well tolerated in solid tumours and phase II studies indicated that it may improve survival in metastatic pancreatic cancers [203]. Promising results for HPV+ cancers have also been obtained: we recently demonstrated that two JAK inhibitors, ruxolitinib and fedratinib, reduced STAT3 and STAT5 phosphorylation, decreased proliferation and induced apoptosis in HPV+ cervical cancer cells [163]. 

These studies suggest that the targeting of JAKs may have clinical benefit in many solid tumours, including HPV+ cancers. JAK inhibition has the added benefit of potentially targeting other signalling pathways, such as ERK signalling, which is also highly active in many HPV+ cancers [232]. Furthermore, in contrast to targeting the IL-6 signalling pathway, targeting JAKs may also inhibit STAT signalling downstream of other receptors.

## 7. Conclusions

A growing body of literature highlights the essential role for JAK-STAT signalling in both the productive HPV life cycle and in HPV-associated cancers. These receptor mediated signalling cascades are manipulated by the HPV oncoproteins to re-wire host cell signal transduction. The dependence on these pathways by HPV for replication and cancer cell proliferation and survival offers an opportunity for therapeutic intervention. A number of strategies are currently being employed to develop effective JAK-STAT inhibitors. As clinical trials progress we will determine whether targeting of these crucial pathways offers a clinical opportunity to treat HPV-associated diseases.

## Figures and Tables

**Table 1:** Current therapeutic strategies to inhibit the JAK/STAT pathway. Details are discussed in the text. PROTAC, proteolysis targeting chimera; HNSCC, head and neck squamous cell carcinoma; mAb, monoclonal antibody; RA, rheumatoid arthritis, FDA, Food and Drug Administration; IBD, irritable bowel disease.

Therapeutic	Target/Mechanism	Indication	Regulatory Status	References
OPB-31121	STAT3 dimerisation inhibitor	e.g., Advanced solid tumours	Phase I	[183]
OPB-111077				[184,185]
IST5-002	STAT5 dimerisation inhibitor	e.g., pancreatic cancer	Pre-clinical	[186]
SD-36	STAT3 [PROTAC]	e.g., leukaemias and lymphomas	Pre-clinical	[187]
STAT3 decoy	STAT3 response element from *FOS* gene	HNSCC	Pre-clinical	[188]
STAT5 decoy	STAT5 decoy oligonucleotide	e.g., leukaemias	Pre-clinical	[189]
AZD9150	STAT3 antisense oligonucleotide	e.g., solid tumours, metastatic HNSCC	Phase I/II	[188,190]
T40214	G-quartet oligodeoxynucleotides	e.g., HNSCC, liver cancer	Pre-clinical	[191]
T40231		e.g., HNSCC, prostate cancer	Pre-clinical	[192]
Siltuximab	Anti-IL-6 mAb	e.g., multiple myeloma, solid tumours	Phase I/II	[193,194,195]
Olokizumab		e.g., RA	Phase II	[196]
Tocilizumab	Anti-IL-6R mAb	e.g., RA	FDA approved	[197,198]
Sarilumab				[199]
Olamkicept	Soluble gp130-Fc fusion protein	e.g., RA, IBD	Phase I/II	[200]
Tofacitinib	JAK inhibitor	e.g., RA, psoriasis, myelofibrosis	FDA approved	[201]
Ruxolitinib			FDA approved	[202,203]
Pacritinib			Phase II	[204]
